# Social Media Data for Omicron Detection from Unscripted Voice Samples

**DOI:** 10.1101/2022.09.13.22279673

**Published:** 2022-09-18

**Authors:** James Anibal, Adam Landa, Hang Nguyen, Alec Peltekian, Andrew Shin, Anna Christou, Lindsey Hazen, Miranda Song, Jocelyne Rivera, Robert Morhard, Ulas Bagci, Ming Li, David Clifton, Bradford Wood

**Affiliations:** 1. Center for Interventional Oncology, Radiology and Imaging Sciences, NIH Clinical Center, National Cancer Institute, National Institute of Biomedical Imaging and Bioengineering, National Institutes of Health, 10 Center Dr, Building 10, Room 1C341, MSC 1182, Bethesda, MD 20892-1182 USA; 2. Computational Health Informatics Lab, Oxford Institute of Biomedical Engineering, University of Oxford, Old Road Campus Research Building, Headington, Oxford OX3 7DQ, United Kingdom; 3. Oxford University Clinical Research Unit, Centre for Tropical Medicine, 764 Vo Van Kiet, Quan 5, Ho Chi Minh City, Vietnam; 4. Department of Computer Science, McCormick School of Engineering, Northwestern University, Mudd Hall, 2233 Tech Drive, Third Floor, Evanston, IL, 60208 USA; 5. Department of Philosophy, University of California, Santa Barbara, Santa Barbara, CA 93106-3090 USA; 6. Feinberg School of Medicine, Northwestern University, 420 E Superior St, Chicago, IL 60611 USA

## Abstract

Social media data can boost artificial intelligence (AI) systems designed for clinical applications by expanding data sources that are otherwise limited in size. Currently, deep learning methods applied to large social media datasets are used for a variety of biomedical tasks, including forecasting the onset of mental illness and detecting outbreaks of new diseases. However, exploration of online data as a training component for diagnostics tools remains rare, despite the deluge of information that is available through various APIs. In this study, data from YouTube was used to train a model to detect the Omicron variants of SARS-CoV-2 from changes in the human voice. According to the ZOE Health Study, laryngitis and hoarse voice were among the most common symptoms of the Omicron variant, regardless of vaccination status.^1^ Omicron is characterized by pre-symptomatic transmission as well as mild or absent symptoms. Therefore, impactful screening methodologies may benefit from speed, convenience, and non-invasive ergonomics. We mined YouTube to collect voice data from individuals with self-declared positive COVID-19 tests during time periods where the Omicron variant (or sub-variants, including BA.4/5) consisted of more than 95% of cases.^2,3,4^ Our dataset contained 183 distinct Omicron samples (28.39 hours), 192 healthy samples (33.90 hours), 138 samples from other upper respiratory infections (8.09 hours), and 133 samples from non-Omicron variants of COVID-19 (22.84 hours). We used a flexible data collection protocol and implemented a simple augmentation strategy that leveraged intra-sample variance arising from the diversity of unscripted speech (different words, phrases, and tones). This approach led to enhanced model generalization despite a relatively small number of samples. We trained a DenseNet model to detect Omicron in subjects with self-declared positive COVID-19 tests. Our model achieved 86% sensitivity and 81% specificity when detecting healthy voices (asymptomatic negative vs. all positive). We also achieved 76% sensitivity and 70% specificity separating between symptomatic negative samples and all positive samples. This result showed that social media data may be used to counterbalance the limited amount of well-curated data commonly available for deep learning tasks in clinical medicine. Our work demonstrates the potential of digital, non-invasive diagnostic methods trained with public online data and explores novel design paradigms for diagnostic tools that rely on audio data.

## 1. Introduction

In this work, we used public online data from social media to boost the diagnostic potential of the human voice. Instant assessment was performed for the presence of the COVID-19 Omicron variant using a deep convolutional neural network (CNN) model trained on unscripted audio samples from YouTube videos. SARS-CoV-2 is routinely detected and confirmed through polymerase chain reaction (PCR) using nasal or throat swabs; however, the turnaround time, cost, and resources can pose a challenge for broad-scale rapid testing in some settings. Resourcelimited settings for definitive testing might benefit from serial screening methods to triage limited testing resources. Invasive home testing methods have also been developed, but can require expensive reagents and laboratory expertise, further restricting accessibility in lowresource settings. Moreover, these tests still do not offer the practicality of immediate results, which have become increasingly necessary as societies move towards “living with COVID”.^5^ Serial testing practices are already common, where reasonably sensitive at-home antigen test screening is followed sequentially by more-specific PCR confirmation. Instant, non-invasive, and sensitive “pre-screening” tests might be useful if immediately available to suggest subsequent confirmatory testing, or to track spread of variants with unique audio phenotypes. Prior AI methods have been unable to successfully detect pre-Omicron variants from unscripted or scripted human voice alone, or were otherwise unsuitable for deployment (e.g., very limited training data, poor generalization).^6^

Omicron, however, more commonly affects the upper airway, sinuses, and hypopharynx than prior variants, often resulting in voice changes without a cough.^7^ This represents an opportunity for more specific targeting by AI methods if robust datasets were available. Worldwide, there are over 3.6 billion users of various social media platforms, and that number is expected to be above 4.4 billion by 2025.^8^ On YouTube alone, over 500 hours of video are uploaded to the platform every minute.^9^ Much of this widely accessible data is ignored but may be readily available to researchers or developers through the advanced programming interfaces (APIs) provided by the social media companies. Such data provides an accurate portrayal of noisy, unscripted “real-world” data, whose broad diversity supports generalizability. Similar data for training and validation may support “pre-screening” deployment settings, such as a smartphone application.

Our deep learning model was trained from this freely accessible data. However, sequencing was not performed, and annotation of training data was based upon prevalence assumptions and selfdeclaration. However, these same limitations contribute to the practicality and cost-effectiveness of the approach. Such models may have non-invasive serial pre-screening implications in settings with unmet needs or in under-resourced populations, with limited access to conventional testing. Early detection of phenotypic shifts or geographic migration could have critical impact in precisely such settings, where low rates of vaccination may facilitate emergence or spread of novel variants. If validated and deployed successfully, AI-based pre-screening tools using voice data alone could be instant, accessible, and cost-effective. Other pre-screening methods that rely upon lab results are slower and less accessible than digital or automated solutions. Digital voice models may be flexibly deployment in a broad array of real-world settings.

There are numerous barriers to realistic deployment of AI models for COVID-19 diagnostics. Prior attempts have frequently been trained on extremely limited datasets, resulting in overfit models that do not generalize, with limited performance and impracticable translation.^10^ Existing models for acoustic, AI-driven diagnostics have also been limited due to a reliance on short, structured samples collected in sterile scripted environments, which are not reflective of real-world settings. In this report, we offer several contributions toward instant COVID-19 testing and, more broadly, to dataset design and audio-based deep learning methodologies, especially in unscripted settings.

### Contributions:

1. A non-invasive and instant Omicron pre-screening/detection model was developed using training data that included recent subvariants, healthy controls, and other respiratory illnesses. Voice changes were identified in patients with self-declared Omicron. Unscripted voice recordings alone (excluding cough, stridor, etc.) may be sufficient for detecting Omicron COVID-19.
2. An AI system was built with public social media data, with training and validation strategies designed for practical real-world settings. This is in contrast with prior social media AI efforts which simply facilitated narrow tasks that were only useful in similar social media settings for similar users. The system included a model for healthy screening (filtering out healthy voices) that was trained on healthy and omicron data. We also trained a model for symptomatic testing, separating other URI data from omicron data.
3. A large audio training dataset was developed from a diverse array of settings and recordings towards Omicron detection from voice sounds. Our dataset contained over 28 hours of unscripted audio from people posting with self-declared Omicron, which is several orders of magnitude greater than previous efforts.
4. A new framework was designed for rapid diagnostic tools from voice data, emphasizing longer samples and unscripted collection protocols that facilitate stable, real-world deployment. The simple strategy relied on the diversity of the input data to prepare for real-world testing environments more effectively. Improved generalization was shown when compared to the use of short speaking inputs using a standardized script (counting to 20). This testing paradigm may be extendable to future pandemics.

## 2. Related Work

### 2.1 Social Media Data for Diagnosis

Cost-effective data collection, curation, annotation, and augmentation are critical for enabling AI to track or predict illness. Public online sources like social media are expansive sources of information. This freely available data does not rely upon intricate searching mechanisms or filtered, delayed reporting, potentially facilitating earlier detection of disease outbreaks, surveillance, or epidemiology dynamics.

The vast amount of information available on social media has been utilized in several existing diagnostic models. A deep learning model trained on Tweets was more predictive of atherosclerotic heart disease mortality than a model based on conventional mechanistic input combining socio-economic, demographic, and health risk factors (such as diabetes and obesity).^11^ Word frequency analysis can classify the mental health status of Twitter users.^12^ Multi-agent reinforcement learning has been used to extract textual and visual features from Tweets to predict depression.^13^ Recently, multiple types of textual features were leveraged to detect suicide ideation from Tweets using deep learning methods.^14^

Other social media platforms (YouTube, Facebook, etc.) have also been used as sources for data for biomedical applications, though less frequently than Twitter. Novel biomarkers predicting pre-diabetes, depression, and postpartum depression were discovered via statistical analysis of Facebook data.^15,16^ Manual analysis of brief, unstructured home videos on YouTube by non-clinical raters was able to detect and classify autism in children with a high performance, even outside of traditional clinical environments.^17^ Similar to the Twitter analyses, YouTube audio, visual, and search-history data have successfully detected mental illnesses, including depression and OCD.^18,19^ As such, the generalizability of social media-based models is limited mostly by the context of their training.

### 2.1 AI for COVID-19 Testing

AI methodologies have been applied across a variety of COVID-19 datasets aiming to develop deployable systems for instant screening diagnostics. When coughing and breathing changes occur with COVID-19, these symptoms may have unique features that can be used for classification, even when compared to other upper respiratory infections (URIs). Past and current work should, however, be contextualized using the criteria outlined by Han et al., which point out methodological flaws such as mixing training/testing data, exclusion of other URIs (classifying only fully healthy samples and COVID samples), and overfitting on small datasets.^6^ Prior work has also generally lacked stratification by variant (the studies were centered around “COVID” as a whole.

Nonetheless, previous efforts highlight the potential for using voice/audio data as the foundation for an effective diagnostic tool. A CNN-based model trained on forced-cough recordings of patients with and without COVID-19 was able to recognize COVID-19 with 98.5% sensitivity, which increased to 100% in otherwise asymptomatic subjects.^20^ Audio-based technologies using cough sounds have also been deployed on a smartphone app for COVID-19 detection.^21,22,23,24,25^ Additionally, COVID-19 patients have unique time and frequency domain patterns in breath sounds that may empower CNN models.^26^

In a dynamic pandemic such as COVID-19, crowdsourced datasets allow for continuous and focused sample collection. “Coswara” is a database containing a variety of respiratory sound samples of COVID-19, including cough, breath, and scripted voice data.^27^ Volunteers recorded and uploaded samples on a smartphone or computer, and the database divided them into COVID or non-COVID cohorts.^28^ Numerous researchers have used this database to train AI models for detection of COVID-19 based on cough or breath recordings.^22,25,29^ Prior work used data from pre-Omicron variants (including Alpha and Delta), which affected the voice less commonly than Omicron.^1^

Omicron is characterized by milder illness and the absence of a cough and lung changes that were the substrates for a multitude of prior AI-based COVID-19 models. However, physiological changes in speech (such as from Omicron) may occur before symptoms are present or obvious.^1^ Past studies have used machine learning techniques to build models that classify COVID-19 with scripted speech as one input element. Audio recordings from COVID-19 patients helped train a model to automatically stratify patients based on illness severity, sleep quality, fatigue, and anxiety.^30^ A binary classifier was able to differentiate COVID-19 speech from normal speech based on scripted telephone data.^31^ Spectral features of speech alone were assessed in asymptomatic patients with and without COVID-19, yielding a true positive rate of 70%. However, the likelihood of generalization was limited, as the model was trained on samples from only 22 asymptomatic patients that were directed to read the same sentence.

Multiple studies have used the Coswara database to train models to detect COVID-19 from scripted voice, cough and breathing samples. Such algorithms reported an accuracy of 97% on limited binary datasets, which notably excluded other respiratory illnesses.^32,33^ Deep learning models trained on the “Sounds of COVID” dataset showed that voice alone performed poorly on pre-Omicron data (0.61 ROC-AUC), also without other respiratory illnesses.^6^ Further, most studies focus on multi-input models, and do not clarify exact time-frames of voice data analyzed, nor specify the exact or likely variant (or sub-variant) with sequencing or demographic likelihood statistics, which may have a major impact upon training and performance.

## 3. Methods

**Figure 1.**
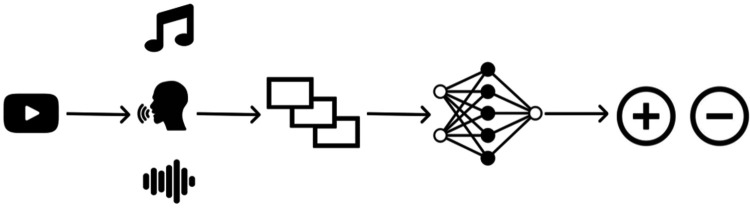
Workflow for COVID-19 detection pipeline. (1) Videos were mined from YouTube; (2) audio was extracted, and the human voice was separated from music and background noise; (3) audio recordings were split into segments and converted to spectrograms; (4) DenseNet model was trained on the spectrograms; (5) trained model was used to predict if samples in a testing dataset were positive or negative for COVID-19.

This study was performed as human subjects research with Institutional Research Board approval and waiver of subject consent. The analysis, training, validation, and testing pipeline for detecting the Omicron variant of COVID-19 using voice recordings mined from YouTube is outlined (Fig. 1). The process included data collection, data preprocessing, and training/validation of the model.

### 3.1 Data Collection

Audio samples were mined from YouTube searches and annotated based upon presumptive correlation with epidemiological data. We separated our data into 4 self-declared, presumptive cohorts:

1. COVID-19 – Omicron variant (presumed by dates)
2. COVID-19 – non-Omicron variant (presumed by dates)
3. Other upper respiratory infections (URIs)
4. Presumably healthy or non-acutely ill subjects.

A series of heuristics were implemented to identify relevant videos. For example, if the user said, “I have COVID” or “I tested positive”, during a time in which Omicron was the dominant variant, the corresponding audio sample was labeled as “Omicron”. For the healthy videos, as there is a nearly infinite number of non-acutely ill speakers on YouTube, we focused on videos which had extremely clear, high-quality audio from one speaker over a long period of time. Each YouTube video was annotated as one of the 4 cohorts and manually verified to ensure accurate labeling. Though the data mining procedure was somewhat standardized, it was not comprehensive, and future studies might benefit from an improved, automated mining procedure to expand the dataset. All Omicron videos were from December 20^th^, 2021 – August 1^st^, 2022. Omicron was designated a “variant of concern” on November 26^th^, 2021 by the WHO.^34^ BA.2 was first identified in the US on December 21^st^, 2021, and Omicron was estimated to be the dominant variant in the US by late December 2021.^35^ Omicron was identified as the dominant variant globally, accounting for > 98% of sequences shared on GISAID after February 2022.^3,36^ The BA.1 and BA.2 lineages were most common between December 2021 – June 2022, with BA.4/BA.5 becoming more prevalent in July 2022.^37^ No sequencing was recorded.

### 3.2 Data Preprocessing

Raw audio samples (extracted from YouTube videos) were noisy, often containing background noise such as music, as well as long periods of silence or low-resolution audio. These were potential sources of confusion for a model aiming to detect disease effects upon spectral features of raw human voice. A preprocessing pipeline with 3 steps was implemented across the cohorts:

1. **Audio Denoising:** Audio quality assessments were performed, and “noisy” sound was removed using semi-supervised machine learning methods developed by Dolby and made available through the Dolby Media APIs.^38^
2. **Removal of Background Noise and Silence:** Background noise was removed via a U-Net convolutional neural network architecture. Extended periods of silence were removed via a voice activity detector that leveraged Gaussian mixture models to identify regions of data wherein the user was not speaking.^39^
3. **Conversion into Mel spectrograms:** Samples were converted into a 3-channel matrix, corresponding to 3 Mel spectrograms generated with different window sizes and hop lengths. Mel spectrograms represent sound as frequency over time, where the frequency values have been converted to the Mel scale (a representation of pitch based on how the human ear perceives loudness). This approach ensured that each channel contained different frequency and time information (similar to resolution in a standard image representation), providing the model with maximal context during training.^40^

### 3.3 Augmentation

In audio-based diagnostics, there is minimal value in positional context. We further assume that, in laryngitis data, long-term dependencies are also limited in value. While past work has shown that not all sounds are “created equal” as disease predictors, relevant digital biomarkers of laryngitis should reasonably have a detectable frequency within a 10 second interval.^41^ Our simple data augmentation strategy relied on the positional invariance of diagnostic speech samples and the reduced need for modeling long-term dependencies in the context of laryngitis. Our aim was to use the natural diversity of speech to enhance the generalizability of our model and reduce the impact of class imbalance. For each audio recording, we considered the set of possible transformations to be the result of dividing the sample recording into segments of length *n* seconds. Time and frequency masking (via SpecAugment) were also applied to each batch prior to input into the DenseNet CNN model.^42^

### 3.4 DenseNet

Convolutional neural networks (CNNs) utilize the convolution operation to model spatial relationships in matrices (e.g., images or spectrograms). These representations, in most cases, are input into a standard feed-forward neural network and mapped onto an outcome or embedding vector. In most cases, the individual layers of a CNN are connected only to the subsequent layer. DenseNet introduced a new framework wherein each layer was connected to all subsequent layers in a “Dense Block”, and there may be multiple Dense Blocks within the same network.^43^ These blocks are connected to each other via convolution and pooling layers which structured the outputs of one block as inputs for the following block. This approach had multiple key advantages for complex tasks, including improved feature propagation and improved feature reuse.

The DenseNet model was chosen due to the scalability of the architecture and high top-1 accuracy value on the complex ImageNet dataset, indicating that this model had learned a generalizable representation of images themselves (key shapes/features). A pre-trained model was chosen based on prior work which reported that CNN models pre-trained on ImageNet achieved superior performance on audio data compared to randomly initialized models.^40,44^ Although DenseNet is used for scalability and high performance, other recent architectures can be used as well, such as Vison Transformer.^45^ Our system does not rely on a single architecture: instead, any improvement in the architectural design our proposed model will also increase the overall outcome.

## 4. Experimental Design

Experiments were performed to assess the potential of social media as a data source for training models to complete pre-screening/diagnostic tasks. We also assessed the generalization capacity gained from using the long, free-response inputs as a component of the data augmentation strategy alongside SpecAugment (compared to short, standardized inputs). Each experiment was run using stratified 6-fold cross validation to determine the potential for generalization. The reported performance metrics are mean values from 6-fold stratified cross validation (Table 2).

### 4.1 Datasets

#### 4.1.1 YouTube Dataset

The YouTube dataset contained 183 videos/subjects with Omicron voices (28.39 hours), 133 videos/subjects with pre-Omicron COVID-19 voices (22.84 hours), 138 videos/subjects with voices from users with other respiratory illnesses (8.09 hours), and 192 videos (33.90 hours) with healthy voices. Statistics for the YouTube dataset are listed in in Table 1.

To the best of our knowledge, the YouTube dataset currently contains the largest amount of voice data (in hours) for COVID-19 generally (all variants), the Omicron variants specifically, and, particularly, upper respiratory infections that were confirmed or self-declared non-COVID. The Coswara dataset contains samples which may be other upper respiratory infections but were not confirmed or self-declared as non-COVID. We also note that within the previously reported “Sounds of COVID” dataset from Han. et al, nearly half the 1,964 negative participants had at least one “COVID” symptom, but multiple of these possibilities were unrelated to the upper respiratory system (e.g., fever, dizziness).^6^ In this study, each participant was instructed to read the same short sentence 3 separate times.^6^

In contrast, the YouTube dataset intentionally contained either self-declared non-omicron illnesses or illnesses that were designated non-omicron based on the date of posting. The samples were selected for the potential to impact the upper respiratory system and strengthen the model as a tool for symptomatic testing with voice data. These included influenza, strep throat, cold, allergy attack, asthma, bronchitis, and others. Prior to training, we applied preprocessing and augmentation strategies to the dataset as described in sections 3.2–3.3.

#### 4.1.2 Coswara

After preprocessing the data to remove samples with more than 50% silence or background noise (section 3.2), the remaining subset of the Coswara database contained 213 Omicron samples (defined using the same date range as the YouTube data), 251 non-Omicron COVID-19 samples, 102 samples from non-diagnosed URIs (COVID-19 not confirmed or ruled out), and 704 healthy samples (on date of access). These scripted samples include audio from counting in English at two rates of speed: normal and fast. Statistics for the Coswara dataset are listed in Table 1.

### 4.2 DenseNet Model Training

For supervised classification tasks involving voice samples, a cross-entropy loss function was used to fine-tune a pre-trained DenseNet. Parameter optimization was performed for learning rate, weight decay, segment length (the length of the *n*-second windows described in section 3.2), and minimum sample length. The final set of parameters used for fine-tuning our model was a learning rate of 1e-5, weight decay of 0.1, a batch size of 64, a segment length of 2.5 seconds, and a minimum sample length of 30 seconds. Early stopping was used to reduce overfitting. To address imbalances in the dataset, each batch was generated by oversampling the minority class. For each sample in the batch, a 2.5 second voice segment was selected randomly from the entire audio recording. We used the same DenseNet architecture when training on the Coswara dataset but froze the base layers and fine-tuned only the classification head. This was done to reduce overfitting on the scripted training dataset.

#### 4.2.1 Healthy Screening

A DenseNet model was trained to perform healthy pre-screening (e.g., an asymptomatic individual, such as prior to attending a sporting event or traveling). Here, the model was trained by simply performing a binary classification task, separating asymptomatic healthy voices from those with omicron.

#### 4.2.2 Symptomatic Testing

A DenseNet model was also trained to perform symptomatic testing. The model was tasked with separating between Omicron samples and other URI samples, aiming to classify users who are presenting with upper-respiratory symptoms

#### 4.2.3 Coswara Experiments

A DenseNet model (classification head only; the base layers were frozen due to overfitting) was fine-tuned on the preprocessed Coswara dataset to complete the tasks described in 4.2.1–4.2.2.

### 4.3 Model Validation

For validation on a blind test dataset, sensitivity and specificity were calculated on a per-sample basis. Each sample was divided into a set of *n*-second segments as described in section 3.3. If a sample could be split into more than one segment, a majority vote was used to assign the final label (“positive” or “negative”). This approach was used to facilitate subsequent real-world deployment, where the user would be prompted to supply at minimum 30 seconds of audio, thereby reducing the risk of incorrect results because of random noise (e.g., misuse of the system) or model failure due to shifts in tone, words/phrases, etc. that could occlude relevant digital biomarkers.

## 5. Experimental Results

### 5.1 YouTube Dataset

Our model was tested on the YouTube dataset using the classification tasks described in sections 4.2.1–4.2.3 (Table 2 for specific results). Notably, we show that voice changes can be used as a predictor for the omicron variant on “real-world” data (mined from YouTube), with a sensitivity of 0.76 and a specificity of 0.70 for the *symptomatic* testing task. This finding suggests that AI models trained on real-world voice data may have relevance beyond identifying asymptomatic healthy subjects. Here, the sensitivity (0.86) and specificity (0.81) were expectedly higher.

### 5.2 Coswara Dataset

For comparison, the same general methodology was applied to the preprocessed Coswara dataset, which consisted of much shorter segments with a standardized script for the participants (counting to 20 in English). Coswara data was used to train a DenseNet model, which was tested on Coswara data subsets. The performance (sensitivity and specificity) was superior for the YouTube dataset versus the Coswara dataset (Table 2). The uniformity of short, scripted data samples may degrade the generalization performance of the model, limiting applications in “real-world” settings.

## 6. Discussion

In this report, we show that:

1. Public online data, including unscripted social media data, has rich public health and epidemiological information that can be utilized in various targeted tasks, even in a pandemic setting. Artificial Intelligence (AI) deep learning models trained from unscripted social media data can be applied in settings that do not involve social media/Internet users.
2. Voice change was a predictor of the Omicron variant (in contradistinction to past variants). Omicron samples were distinguished not only from healthy voices, but also from voices with other URIs/conditions.
3. Models trained on longer, unscripted audio samples achieved superior generalization compared to shorter, and, in some cases, standardized scripted inputs. Lengthy sequential data improved model performance, even for audio diagnostics where there is little need for modeling positional context or long-term dependencies.

We introduced the “YouTube COVID-19 voice dataset”, which contains over one full day of data from audio samples corresponding to the Omicron variant, non-Omicron COVID-19, and healthy controls. The dataset also included over 8 hours of data from other URIs. This is in contrast with other COVID-19 audio datasets such as Coswara, which were noisy and imbalanced (approx. 1 hour of viable Omicron voice data) and contained undiagnosed URI data (unconfirmed COVID negativity). This comparison underscores the value of retrospective data collection from public social media in unscripted real-world settings.

### 6.1 Real-World Deployment

Potential future applications for our model include dataset expansion through improved mining methods, smartphone/web-app testing that can be done at the onset of symptoms, prior to large gatherings, travel, or in other surveillance settings. Furthermore, upfront regular pre-screening in an at-risk population (already being screened with PCR or antigen testing) could be used to define the temporal or geographic dynamics of voice changes from variants as the virus continues to evolve. Since infectivity and transmission may pre-date symptoms, such efforts might facilitate a better understanding of early pre-screening methodologies and approaches.

However, model deployment, stability, and impact currently remain speculative and unproven due to the limited size of samples from other URIs, lack of PCR confirmation testing, lack of SARS-CoV-2 sequencing, and numerous assumptions feeding into ground truth classification. Training data classifications were dependent upon prevalence and demographic assumptions which introduce undefined elements.

## 7. Conclusion

Digital epidemiology is understudied in the context of the vast amounts of public online data and social media data available. The quantity of public data raises new questions in terms of security, regulation, and real-world validation. Social media audio data that is unscripted may inherently be more diverse (and ultimately lead to more generalizable models) than narrow-intent scripted data. Still, the results achieved by this early effort at Omicron detection merit further evaluation with smartphone app deployment, even without ground truth from PCR or sequencing in the training data. Despite these limitations, this work highlights the presentation of laryngitis manifesting with uniquely hoarse voices in patients with the Omicron variant of COVID-19. The deluge of untapped and unscripted audio data on social media may enable new frameworks to facilitate instant pre-testing, all trained with public data.

## Figures and Tables

**Table 1:** Statistics for COVID-19 Sound/Voice Datasets used in this study.

Dataset	COVID-19 Samples (All variants)	Omicron Samples	Other URI (Symptomatic) Samples	COVID-19 total audio	Omicron total audio	URI total audio
YouTube Dataset	316	183	138	**51.23**	**28.39**	**8.09**
Coswara	464	**213**	102	1.92	0.95	0.47

**Table 2:** Model performance on randomly selected test datasets using the unscripted YouTube and scripted Coswara datasets:

Dataset	Task	Sensitivity	Specificity
YouTube	Healthy Screening	0.86	0.81
YouTube	Symptomatic Testing	0.76	0.70
Coswara	Healthy Screening	0.58	0.55
Coswara	Symptomatic Testing	0.52	0.43
